# Acute lung injury and respiratory failure following ammonia gas inhalation: A case report

**DOI:** 10.6026/973206300212473

**Published:** 2025-08-31

**Authors:** Sucheta Aurora, Shahid Patel, Girija Nair, Nikhil Sarangdhar

**Affiliations:** 1 Department of Pulmonary Medicine, DY Patil University, Nerul, Navi Mumbai, India

**Keywords:** Acute lung injury, respiratory, inhalation, radiography, ammonia gas, lungs failure

## Abstract

Acute lung injury due to irritant gases is usually associated with tissue damage extending from the airways to the lung parenchyma and alveoli, causing respiratory failure that may necessitate assisted ventilation. We describe here a case of ammonia gas inhalation leading to acute lung injury and respiratory failure, diagnosed based on a correlation of history, clinical features, imaging and lab parameters and managed with non-invasive pressure support and supplemental oxygen.

## Background:

A 20-year-old male presented to DY Patil Hospital Emergency Department at 8 AM with an alleged history of accidental ammonia gas inhalation from a cold storage facility where he worked the night shift. In the early hours of the morning, he sensed an unpleasant, irritating, noxious odor accompanied by a burning sensation in his respiratory tract, followed by a bout of giddiness. He was found unconscious by colleagues, who revived him and brought him to the hospital. At presentation, he complained of nausea, vomiting, redness and irritation in eyes and throat, chest pain and breathlessness. He was a non-smoker with no known comorbidities. On examination, the patient was febrile with a pulse rate of 110 beats per minute, blood pressure of 100/70 mmHg and a respiratory rate of 22 breaths per minute. His oxygen saturation (SPO2) was 95% on room air. Respiratory system examination was unremarkable. Chest radiography was normal (Figure 1 - see PDF). ECG showed tachycardia with a normal sinus rhythm. However, his condition deteriorated two hours later, becoming drowsy and tachypneic with a respiratory rate of 28/min and oxygen saturation of 90% on room air. On auscultation, diffuse rhonchi and crackles were noted. Serum ammonia was elevated at 128 mg/dL. His arterial blood gas (ABG) analysis on room air showed pH 7.416, pCO2 44.6 mmHg, pO2 78.4 mmHg, HCO3 27.9 mmol/L and SPO2 90.3%. He was started on supplemental oxygen via a face mask and maintained an oxygen saturation of 96% on 5 L O2. ABG on 5 L O2 showed pH 7.37, pCO2 42.3 mmHg, pO2 81.9 mmHg, HCO3 24.2 mmol/L and SPO2 94.8%. Bilateral rhonchi and crackles persisted on auscultation and chest radiography revealed bilateral infiltrates (Figure 2 - see PDF). Despite oxygen therapy, he remained tachypneic and repeat ABG on 5 L O2 revealed pH 7.32, pCO2 47.4 mmHg, pO2 90.9 mmHg, HCO3 28.3 mmol/L and SPO2 92.7%. The patient was shifted to the ICU and placed on non-invasive ventilation with pressure support. Nebulized bronchodilators, intravenous corticosteroids, injectable antibiotics, intravenous furosemide and methylprednisolone were administered. Subsequently, he developed a fever with a temperature of 101°F. A follow-up ABG five hours later revealed metabolic acidosis with pH 7.024, pCO2 35.9 mmHg, pO2 204 mmHg, HCO3 9.1 mmol/L and SPO2 98.8%. Injectable bicarbonate was administered and he responded well, with improvements in both clinical and biochemical parameters. High-resolution computed tomography (HRCT) revealed multiple centrilobular opacities involving the anterior and posterior segments of the right upper and lower lobes, along with small fibrotic bands and pleural thickening in the posterior segment of the left lower lobe (Figure 3 - see PDF). A 2D echocardiogram was normal. Flexible bronchoscopy with lavage was advised, but the patient declined consent. Despite this, he continued to show clinical improvement. After three days, chest radiography showed clearing of infiltrates (Figure 4 - see PDF) and ABG parameters normalized. Fever and tachypnea resolved and oxygen supplementation was no longer required.

Spirometry with diffusion capacity testing showed FEV1/FVC at 66.12%, FEV1 at 82.5%, indicating mild obstruction with good bronchodilator reversibility and a moderately reduced diffusion capacity (55%). The patient was discharged after four days on formoterol and budesonide via metered-dose inhaler with a spacer, along with tapering doses of oral methylprednisolone. One-month post-discharge, chest radiography was normal and spirometry showed improvement with normal FEV1/FVC and improved diffusion capacity (DLCO) to 75%. Ammonia (NH_3_) is a colorless gas with a pungent odor, extensively used in industries such as agriculture, refrigeration and manufacturing. Biochemically, ammonia is a weak base (pKa 9.25) that dissolves readily in water to form ammonium hydroxide (NH_4_OH), a highly alkaline solution [1, 2]. Upon inhalation, ammonia contacts the moist mucous membranes of the respiratory tract, dissolving rapidly and exerting a caustic effect, leading to direct chemical injury. This injury results from the denaturation of proteins and disruption of membrane lipids, increasing cell permeability and causing tissue necrosis [3, 4]. Ammonia exposure exacerbates tissue damage by generating reactive oxygen and nitrogen species, contributing to oxidative stress and further impairing cellular function [5]. The degree of injury varies with the concentration as well as length of exposure. Low concentrations only have irritation effects on the eyes, nose and throat. Increased concentrations affect the trachea, bronchi and alveolar structures and may cause pulmonary edema and altered gas exchange [6, 7]. Severe injuries from smoke inhalation trigger an inflammatory reaction, which leads to the release of cytokines and chemokines aggravating tissue injury and contributing to the development of acute respiratory distress syndrome (ARDS) [8]. Acute exposure to high concentrations of ammonia may lead to acute respiratory injury but also to long term impairment of respiratory function [12]. Prompt identification and treatment prevent the development of severe complications of ammonia-induced lung injury. Supplemental oxygen and non-invasive ventilation are provided to stabilize respiratory function and reduce inflammation [9]. The mechanisms discussed help in providing appropriate treatment in time and minimizing long-term sequelae of respiratory organs [10].

## Discussion:

Our case involved a young, healthy male with no prior comorbidities who experienced acute high-dose occupational ammonia inhalation exposure while working in a confined environment. He initially presented with respiratory distress and normal chest radiography findings, which later worsened, leading to significant hypoxemia. The patient's clinical course deteriorated, requiring non-invasive ventilation and oxygen support. Follow-up evaluations showed lung function abnormalities, diffusion capacity impairments, radiological changes and abnormal blood gas levels. However, a near-total improvement was obtained with normalization of radiographic features, lung function and blood gases but with a minimal impairment in diffusion capacity.

The striking feature in this case is the separation between the early respiratory symptoms and the presence of radiographic findings, which indicates the onset of radiological abnormality is somewhat delayed despite severe clinical illness. Early and sustained intervention with non-invasive ventilation and careful monitoring resulted in rapid clinical improvement and stabilization within a month of discharge. The favorable outcome in this case underscores the importance of prompt clinical suspicion and swift management strategies, even in resource-limited settings. Early recognition, triage and timely respiratory support can be critical in mitigating the severe consequences of ammonia inhalation injury [11]. Although oxygen therapy is the cornerstone in the management of acute respiratory distress, prolonged high concentrations of supplemental oxygen are potentially dangerous and pose a risk for oxygen toxicity that may cause further lung injury. This can present as alveolar injury, pulmonary edema and inflammation as a result of the excessive generation of reactive oxygen species [10]. In our patient, careful tapering of oxygen supplementation and monitoring of blood gases were crucial in minimizing this risk while ensuring adequate oxygenation. Balancing the therapeutic benefits of oxygen therapy with the potential for toxicity remains a critical aspect of managing inhalation injuries like ammonia exposure.

## Conclusion:

This case illustrates the severe impact of acute occupational ammonia exposure and highlights the potential for full recovery with timely and appropriate management. Effective triage, early respiratory support and diligent follow-up are essential in achieving favorable outcomes even in challenging clinical scenarios.

## References

[1] Tonelli AR (2009). Burns..

[2] Zhang F (2015). Burns..

[3] Sobonya R (1977). Hum Pathol..

[4] Brautbar N (2003). Arch Environ Health..

[5] Jones SW (2017). Clin Plast Surg..

[6] Pirjavec A (2009). J Trauma..

[7] Lemire P (2020). Environ Int..

[8] Pangeni RP (2022). Ann Med Surg (Lond)..

[9] Kim I (2022). Kosin Med J..

[10] Vadysinghe AN (2021). Am J Forensic Med Pathol..

[11] Hua Y (2024). J Burn Care Res..

[12] Leduc D (1992). Thorax..

